# Editorial: Chromatin structure and function

**DOI:** 10.3389/fgene.2023.1140534

**Published:** 2023-02-07

**Authors:** Laxmi Narayan Mishra, Christophe Thiriet, Dileep Vasudevan

**Affiliations:** ^1^ Albert Einstein College of Medicine, Bronx, NY, United States; ^2^ Université de Rennes 1, CNRS-UMR6290 Institut génétique et développement de Rennes (IGDR), Rennes, France; ^3^ DBT-Institute of Life Sciences, Bhubaneswar, India

**Keywords:** chromatin, histone, histone posttranslational modifications, chromatin remodeling, DNA damage repair

Chromatin dynamics influence DNA-dependent processes such as transcription, repair, replication, and recombination (Hübner and Spector, 2010; Galvani and Thiriet, 2015). Because disorganized chromatin affects gene expression and eventually leads to disease onset, scientists are eager to learn more about the roles of histone PTMs, DNA methylation, and chromatin remodeling factors in chromatin dynamics. In eukaryotes, how genes are packaged in chromatin determines whether or not the genes can be expressed to produce the encoded product. Typically, DNA-binding factors cannot access DNA within the nucleosome, and nucleosomes must be disassembled for them to gain access to the underlying DNA (Mishra and Hayes, 2018; Sundaram and Vasudevan, 2020). Various high-throughput technologies have emerged to aid researchers in understanding chromatin structure and function (Marr et al., 2022). This Research Topic contains numerous articles on chromatin dynamics, transcription, DNA damage repair, and drug resistance.

In their study, Vinaychandran and Bhargava describe how the structural characteristics of nucleosomal DNA affect transcription factor binding and effective translation. Shi et al. reviewed recent advances in determining chromatin dynamics and their modulation by factors such as PTMs, histone variant incorporation, and effector protein binding. Seharawat et al. discuss how histone PTMs affect nucleosome structure and regulate chromatin accessibility in a review. Pavlenko et al.’s review article summarizes current knowledge on the functions of lysine-specific demethylase-5 and focuses on molecular interactions and their potential implications. They also bring unanswered questions about histone demethylation that require the scientific community’s attention to understand it fully.

Genome attacks are common throughout cell life, and various factors can cause DNA damage. When a DNA lesion occurs, the cell repairs it to preserve the genetic material’s integrity. Because the genome has been condensed into chromatin, the repair must occur within the context of chromatin structure to access and repair damaged DNA (Hauer and Gasser, 2017). Bisht et al. in their research article, show that the interaction of SMARCAL1 and BRG1, two chromatin remodeling factors that collaborate in the promoter region during double-stranded DNA repair, is dependent on their ATPase activity. Based on BRCA1 and 53BP1 abundance and organization, Abate and Hendzel’s study demonstrates the presence of multiple classes of DNA double-strand break (DSB) repair compartments. Roemer et al. discuss constitutive heterochromatin accessibility. They studied the chromocenter concentrations and diffusion of several DSB sensors, mediators, and effector proteins in mice without DNA damage using fluorescently labeled proteins involved in DNA damage detection and repair. In their review, Aricthota et al. discuss the role of histone acetylation in altering chromatin organization and promoting the recruitment of DSB repair proteins to DNA damage sites.

On the other hand, nucleosome assembly is required to restore the native nucleosomal template and the correct epigenetic landscape, which is most visible during DNA replication. The newly synthesized DNA must be packaged in a consistent, complementary, and epigenetically tagged fashion. At the replication fork, a highly orchestrated mechanism not only creates templates and produces an identical copy of DNA but also removes nucleosomes in front and reassembles histones into nucleosomes behind (Verreault, 2000). Zhao et al. reconstituted nucleosomes *in vitro* using the nucleosome positioning sequence Widom 601 and proposed a chemical-kinetic model of nucleosome assembly and disassembly using precise biophysical methods such as FRET and FTS assays. Gene regulation, recombination, and other fundamental processes rely on large-scale chromatin interactions, including chromosome interactions. Krajewski discusses how bulky post-translational histone modifications like ubiquitination, internucleosomal dynamics, and DNA stress work together to functionalize nucleosomes in a large nucleosome array in his hypothesis and theory paper.

Methylation of DNA regulates gene expression by generally turning the gene off (Moore and Fan, 2013). Li et al. discuss the limitations of identifying N6-methyladenine, a poorly studied DNA methylation in eukaryotes. They also discuss the potential applications of this recently discovered DNA modification. In a systematic review, Rawat et al. discuss the coexisting mutations and gene expression trends associated with K13-mediated artemisinin resistance in *Plasmodium falciparum*. They analyzed a large dataset of single nucleotide polymorphisms (SNPs) to determine the prevalence, geographic distribution, and coexistence patterns of genetic markers associated with artemisinin resistance.

Overall, the breadth of the articles in this Research Topic demonstrates the remarkable progress being made in understanding the critical roles of chromatin structure in transcription, replication, and DNA damage repair. These articles also raise numerous unanswered questions that must be addressed in the near future to fully understand the disease biology associated with altered chromatin structure.
